# Device Therapy in Cardiac Sarcoidosis: Current Review, Challenges, and Future Prospects

**DOI:** 10.19102/icrm.2024.15115

**Published:** 2024-11-15

**Authors:** Mohamed ElRefai, Christina Menexi, Paul R. Roberts

**Affiliations:** 1 Cardiology Department, University Hospital of Cambridge, Cambridge, UK; 2 Cardiology Department, Essex Cardiothoracic Centre, Essex, UK; 3 Cardiology Department, University Hospital of Southampton NHS Foundation Trust, Southampton, UK; 4 Cardiology Department, University of Southampton, Southampton, UK

**Keywords:** Cardiac sarcoidosis, leadless pacemaker, subcutaneous ICD, sudden cardiac death

## Abstract

Sarcoidosis is a complex disease characterized by inflammatory granulomas that can affect various organs, including the heart. The diagnosis of cardiac sarcoidosis poses challenges, and current criteria involve the use of advanced imaging techniques and histological confirmation. Clinical manifestations of cardiac sarcoidosis vary widely, ranging from heart block to ventricular tachycardia and heart failure. Sudden cardiac death (SCD) is a significant concern, and implantable cardioverter-defibrillators (ICDs) are recommended for preventing SCD in high-risk cases. However, some patients with cardiac sarcoidosis do not meet the current guidelines for ICD implantation, leaving them at risk. Traditional transvenous ICDs are associated with complications, especially in immunosuppressed patients. The subcutaneous implantable cardioverter-defibrillator (S-ICD) offers a potential solution, as it avoids vascular complications and reduces the risk of infections. However, concerns regarding inappropriate shocks and the lack of pacing therapy limit its widespread use. Leadless pacing combined with S-ICD represents a potential novel approach to managing cardiac sarcoidosis patients. Ongoing human clinical trials are expected to shed light on the safety and efficacy of this combined therapy. Cardiac sarcoidosis patients, who have been underserved by traditional device therapies, may benefit from this personalized approach. Further research is needed to guide the management of SCD risk in this population.

## Introduction

**Figure 1:**
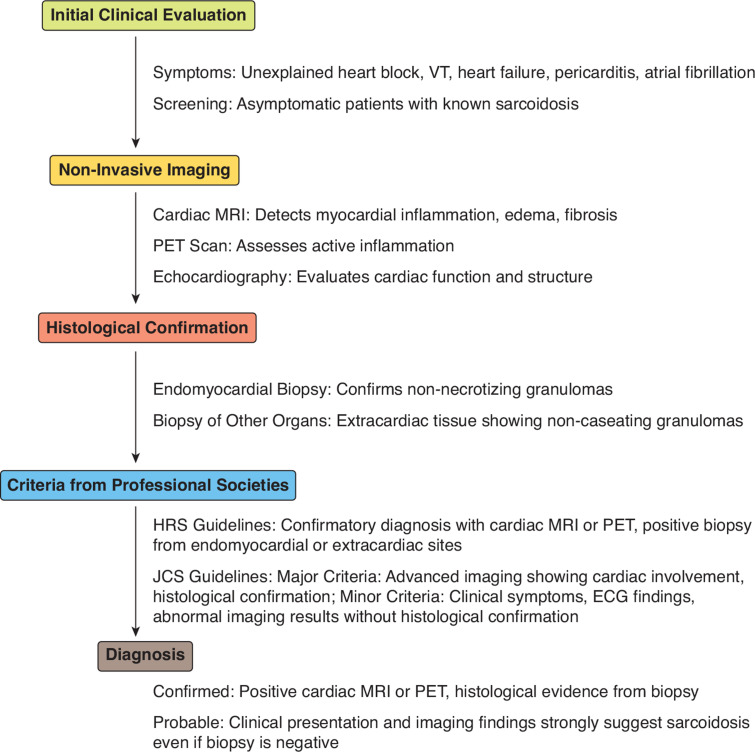
Flowchart for the diagnosis of cardiac sarcoidosis. *Abbreviations:* ECG, electrocardiogram; HRS, Heart Rhythm Society; JCS, Japanese Circulation Society; MRI, magnetic resonance imaging; PET, positron emission tomography; VT, ventricular tachycardia.

Sarcoidosis remains a significant health condition with an undetermined etiology. It is thought to be a consequence of an immunological response triggered by exposure to environmental factors in genetically predisposed subjects.^1^ It is characterized by inflammatory granulomas that can either heal spontaneously or end up in irreversible fibrosis. Cardiac sarcoidosis is characterized by the infiltration of the cardiac tissue with the non-necrotic granulomas characteristic of this condition.^1^ The diagnosis of cardiac sarcoidosis remains a challenge, and there are several criteria proposed by different professional societies for the diagnosis of cardiac sarcoidosis that are currently used in practice. These criteria involve the use of advanced imaging techniques like cardiac magnetic resonance imaging (CMRI) and positron emission tomography (PET) scanning, and some require proof of histology on endomyocardial biopsy for a confirmed diagnosis of cardiac sarcoidosis **(Figure 1)**.^2–4^

Cardiac sarcoidosis can present itself in a multitude of clinical manifestations, depending on the location and the extent of cardiac involvement as well as the activity state of the disease. An otherwise unexplained high-grade atrioventricular (AV) block is quite a common presentation of the disease. Ventricular tachycardia (VT) is also a common occurrence that can manifest either in the inflammatory or the fibrotic phase of the disease. The widespread involvement of the myocardium can manifest in symptoms and signs of heart failure. In addition, the involvement of valvular tissue can result in different grades of valve stenosis or regurgitation. Cardiac sarcoidosis less likely presents with atrial fibrillation, angina-like chest pain, or in the form of unexplained pericarditis. Cardiac sarcoidosis can be clinically silent and only detected on routine screening. Sadly, it can present itself as an unexpected sudden cardiac death (SCD) and is diagnosed in these cases only at autopsy.^1^

Several cohort studies have reported a 5-year survival rate of >90% and a 10-year survival rate of 80%–90% in cardiac sarcoidosis. The overall prognosis relies on the degree of cardiac involvement as well as the manifestation of the disease, where sustained VT or heart failure denotes a worse prognosis. SCD, mostly attributed to fatal arrhythmia, remains the predominant cause of death in cardiac sarcoidosis.^5^

The mainstay of treatment in cardiac sarcoidosis is immunosuppression with corticosteroids as a first-line agent. Other immunosuppressive agents, such as methotrexate, azathioprine, mycophenolate mofetil, leflunomide, and cyclophosphamide, can be used as second-line or sometimes third-line agents, while biologic anti-tumor necrosis factor can be used when others have failed.^1^

The focus in treating this condition is the prevention of ventricular arrhythmias and SCD. Anti-arrhythmic medications as well as catheter ablation have been used for the treatment of ventricular arrhythmias in cardiac sarcoidosis with unpredictable efficacy and common recurrences of the arrhythmias.^6^ Implantable cardioverter-defibrillators (ICDs) are a well-established therapy option for the prevention of SCD in cardiac sarcoidosis.

It is challenging to risk-stratify cardiac sarcoidosis patients owing to the complexity and dynamicity of their underlying pathophysiology. A reduced ejection fraction was previously proposed as an indicator of a high risk of ventricular arrhythmias in cardiac sarcoidosis patients. However, ventricular function might improve over time with treatment, and a history of syncope and the development of AV block are now of greater value in identifying patients at a higher risk of ventricular arrhythmias and SCD.^7,8^ Active inflammation seen on cardiac PET scans and the presence of fibrosis on CMRI are also strong predictors of ventricular arrhythmias and SCD, regardless of the ejection fraction.^9,10^ There is a greater tendency for basal septum involvement in the inflammatory process in cardiac sarcoidosis, which may explain the association of AV block and an increased risk of ventricular arrhythmias.^7^ While the active inflammation usually responds well to steroids, it can result in fibrosis and scarring, which forms a substrate for re-entrant ventricular arrhythmias.

The conjoint 2017 American Heart Association (AHA)/American College of Cardiology (ACC)/Heart Rhythm Society (HRS) guidelines give a class I indication for ICD implantation in cardiac sarcoidosis in the event of a prior aborted cardiac arrest, documented spontaneous sustained VT, or a left ventricular ejection fraction (LVEF) of ≤35%, while they give a class IIa indication for such (1) in patients with an LVEF of >35% and an indication for a permanent pacemaker, (2) in the presence of a history of syncope compatible with an arrhythmogenic etiology, (3) following the induction of a sustained ventricular arrhythmia during an electrophysiology study, or (4) in patients with an LVEF of >35% with evidence of myocardial scar or the presence of extensive scarring detected by CMRI or PET scans.^11^ The 2022 European Society of Cardiology (ESC) guidelines give a class I indication for ICD implantation in the event of a previously aborted cardiac arrest, in the context of documented spontaneous sustained VT, or in patients with an LVEF of ≤35%, while a class IIa indication is given (1) in the presence of an indication for a permanent pacemaker in patients with an LVEF of >35%, (2) in the context of an inducible sustained monomorphic ventricular arrhythmia during an electrophysiology study in patients with an LVEF of 35%–50% and minor late gadolinium enhancement (LGE) at CMRI, (3) or in patients with an LVEF of >35% with significant myocardial LGE at CMRI following resolution of acute inflammation **(Table 1)**.^12^

## Challenges and future prospects

Most patients with clinically manifested cardiac sarcoidosis fit within the guidelines criteria for the implantation of ICDs. However, some cardiac sarcoidosis patients do not have indications for ICD implantation as per the current guidelines and subsequently are not offered this potentially lifesaving therapy.^13^ Nordenswan et al. investigated the incidence of SCD and life-threatening arrhythmias in patients with manifest cardiac sarcoidosis with and without indications for an ICD. The authors studied a cohort of 398 (193 definite and 205 probable cardiac sarcoidosis) patients between 1997 and 2017 in Finland. Patients with and without ICD indications (as per the HRS guidelines) at presentation were identified, and the incidence of SCD and sustained VT events was recorded for a median follow-up period of 5 years. A total of 339 patients (85%) had an indication for ICD implantation, while 59 patients (15%) did not. The 5-year risk of SCD or sustained VT was 24% in the entire cohort and 12% in patients with no indication for an ICD, respectively. The cumulative 5-year incidence of SCD was 10.7% (95% confidence interval [CI], 7.4–15.4) in patients with ICD indications versus 4.8% (95% CI, 1.2–19.1) in those without. In patients without ICD indications, the 5-year incidence of SCD, sustained VT, and emerging indications for ICD during follow-up was 53% (95% CI, 39.5%–70.9%). The authors concluded that the guidelines, in their current form, fail to identify cardiac sarcoidosis patients who are truly at low risk of SCD and who would not benefit from ICDs. They also suggested that all patients with clinically manifested cardiac sarcoidosis should be considered for defibrillator protection until further research reveals a truly low-risk population.^13^

However, ICDs do not come without risks. Traditional ICDs employ transvenous (TV) leads for rhythm discrimination and delivery of defibrillation shock therapy and thus are associated with potential complications, which can be categorized as complications related to the invasion of the vascular space. Potential complications include both those that occur at the time of implantation, such as pneumothorax and cardiac tamponade due to traumatic placement of the lead(s), and long-term complications like device infection—a real concern in cardiac sarcoidosis patients on chronic immunosuppressive therapy. Infection of the device may progress into sepsis and/or infective endocarditis with potentially fatal consequences.^14,15^ Transvenous ICD (TV-ICD) lead longevity is also a significant issue, with the annual rate of TV-ICD lead defects requiring intervention increasing with time and reaching 20% in 10-year-old leads.^14^ The estimated TV-ICD lead survival rates at 5 and 8 years are just 85% and 60%, respectively.^14^ Additionally, ICD leads that remain in the vasculature for many years may, ultimately, compromise flow or cause obstruction. The complications of TV-ICDs in cardiac sarcoidosis patients appear to be even more common than in the general ICD population. In some studies, up to 24% of cardiac sarcoidosis patients who have had TV-ICDs implanted have been reported to receive inappropriate therapies, while 15% may have complications such as infections and lead fracture or dislodgement.^16,17^

The subcutaneous ICD (S-ICD) was designed to avoid complications of the TV-ICD by using an extra-thoracic approach. It involves an electrically active can and a single subcutaneous lead containing two sensing electrodes. The components of this device do not enter the heart or vascular system and are not exposed to the hostile environment of the vessels or the repetitive contractions of the ventricle. Although the S-ICD leads are more exposed to the musculoskeletal movements than the TV leads, they are larger and therefore more robust than the TV leads. S-ICD therapy therefore avoids many of the complications that have been linked to TV-ICDs. However, despite S-ICDs being a well-established therapy option backed up by the guidelines for patients requiring defibrillation protection,^12^ their full potential is still not fully explored. In fact, we have not found any published literature on the use of S-ICDs in cardiac sarcoidosis.

### Subcutaneous implantable cardiac defibrillators in cardiac sarcoidosis

S-ICDs can represent a modern solution that can theoretically tip the balance and allow us to expand the indications of defibrillation protection in cardiac sarcoidosis patients without increasing ICD-related complications. The ultimate goal is to lower the rates of morbidity and mortality in this population.

Being an extravascular device, S-ICDs are associated with lower rates of lead-related complications, both in the acute and the long term.^18^ This is particularly relevant in patients requiring decades of defibrillation therapy, such as the relatively younger population of cardiac sarcoidosis patients. S-ICDs also carry no risk of infective endocarditis, which is of paramount relevance in cardiac sarcoidosis patients who are at an elevated risk of infection owing to the use of (often multiple) immunosuppressive agents for the management of the underlying disease activity.

Despite advancements in detection algorithms, however, S-ICDs are still associated with higher rates of inappropriate shocks in the general ICD population.^18^ The perceived heightened risk of inappropriate shocks, together with the lack of data on S-ICD screening outcomes in the cardiac sarcoidosis population, can dissuade clinicians from considering S-ICD therapy in this patient group, particularly when it has been reported that these patients experience higher rates of inappropriate shocks than the general ICD recipient population.^19^ Furthermore, cardiac sarcoidosis patients not uncommonly can present with monomorphic VT that could be treated with painless antitachycardia pacing (ATP) therapy, which is a treatment modality not offered by S-ICDs. However, the main argument against the use of S-ICDs in cardiac sarcoidosis patients and perhaps one of the main reasons why the use of S-ICDs in this patient group has not been explored further is the fact that, unlike traditional TV-ICDs, S-ICDs cannot provide pacing therapy. This is particularly relevant in cardiac sarcoidosis patients who have a high incidence of conduction system disease requiring pacing therapy.^1^ Complete heart block is the most common finding in patients with cardiac sarcoidosis and can be present in up to 30% of these patients. Lower degrees of AV block as well as intraventricular conduction defects are also not uncommon findings in these patients.^1^

### Extravascular implantable cardiac defibrillators

Extravascular ICDs (EV-ICDs) are a newer type of ICD where the leads are implanted extravascularly, underneath the sternum, reducing risks such as vascular injuries and infections associated with traditional ICDs. These devices are implanted in the left axillary space, maintaining the size, shape, and longevity of traditional ICDs while offering defibrillation protection, ATP, and backup pacing. Compared to S-ICDs, EV-ICDs provide additional benefits. These include ATP capabilities, which S-ICDs lack, allowing them to terminate ventricular arrhythmias without delivering shock therapy. In addition, they are overall of a smaller size, comparable to the generator sizes of the TV-ICDs. EV-ICDs were designed to combine the best features of both traditional ICDs and S-ICDs while mitigating their limitations. Friedman et al. investigated the efficacy and safety of the EV-ICD in a study involving 356 patients. The device achieved a successful defibrillation rate of 98.7%, while safety endpoints showed that 92.6% of patients were free from major complications at 6 months. The study concluded that EV-ICDs are safe and effective for detecting and terminating ventricular arrhythmias.^20^ While the results are promising, real-world data are awaited to further validate the findings and assess the performance of the device in broader clinical practice.

### Leadless pacing

While TV pacemakers are well-established solutions for the management of bradycardia, it has been shown that almost 90% of their complications are related to the presence of endovascular leads and device pocket issues, such as erosion and infection.^15,21,22^ Leadless pacing (LP) is a well-established modality for providing pacing therapy, particularly when TV pacing is not feasible or desired. There are currently a few published reports with recommendations for indications for LP therapy as opposed to the traditional TV pacing, such as the national expert consensus of the Austrian Society of Cardiology,^23^ the recommendations of the expert opinion of the working group on LP of the Polish Cardiac Society,^24^ and the UK Expert Consensus Statement for the Optimal Use and Clinical Utility of Leadless Pacing Systems.^25^ Overall, expert opinions favor the use of LP in young patients and in patients who are immunocompromised. These recommendations would favor the use of LP in cardiac sarcoidosis patients. However, previous studies showed that advanced conduction system disease was also a significant predictor of appropriate ICD therapy.^7,16^ This was highlighted in a previously published editorial by Heck and Roberts, implying that advanced conduction system disease is likely to be a surrogate marker for a more extensive granulomatous infiltration of the myocardium and subsequently a greater risk of ventricular arrhythmias. The authors of the editorial also raised the question as to whether patients with sarcoidosis and pacing indications should have a primary-prevention ICD implanted at the outset.^26^ Currently, guidelines recommend offering defibrillation therapy for cardiac sarcoidosis patients who develop a pacing indication.^11,12^ This is why leadless pacemakers, on their own, are not a suitable management option for these patients despite their potential benefits.

It is also important to highlight some other potential limitations to the use of LP therapy as the first approach in cardiac sarcoidosis patients. Pacing indications in cardiac sarcoidosis patients are most likely due to high-grade AV block, favoring dual-chamber pacing as opposed to single-chamber (VVI) pacing, making VVI LP less ideal. However, with the current advances in LP technologies, such as the atrial sensing/ventricular pacing capabilities of Micra™, a leadless pacemaker developed by Medtronic (Minneapolis, MN, USA), and the introduction of AVEIR™, the dual-chamber LP system developed by Abbott (Chicago, IL, USA), LP can be used to deliver dual-chamber pacing, similar to TV pacing systems. Also, in the presence of a pacing indication with the concomitant presence of left ventricular (LV) impairment, guidelines recommend offering patients pacing modalities that avoid the possibility of pacemaker-induced deterioration of LV function, such as biventricular or conduction system pacing, especially those with an anticipated high burden of pacing requirement. In their current form, leadless pacemakers do not provide biventricular or physiological pacing, although further advances in the technologies are eagerly anticipated to overcome this significant limitation.

### Combined device therapy

Combining an S-ICD with a leadless pacemaker could yield a viable therapy option for patients who would benefit the most from the absence of intravascular leads. This represents a novel concept for the management of such patients in whom device therapy could be further personalized to current and future needs. However, this would essentially require reliable device–device communication. The safety and efficacy of S-ICD–leadless pacemaker device-to-device communication has been evaluated previously in animal studies and was first reported in 2016.^27^ Since then, larger animal studies have been published evaluating the performance of combining LP with an S-ICD in acute as well as chronic (3 months) animal implants. The results of the evaluations were promising, showing stable communication thresholds between the devices throughout the 3-month study.^28^ Although animal model evaluations can be a useful surrogate when human evaluation is not feasible or ethical, animal models cannot fully replicate human anatomy.

There is a paucity of literature on the use of combined leadless and S-ICD device therapy in human subjects, and published papers are mainly limited to case reports.^29–35^ In a case series reported by Calvagna and Valsecchi, a total of 13 patients, 11 with prior TV-ICD infection and 2 with high-risk features for infection, were implanted with both S-ICDs and leadless pacemakers. Both procedures were done in the same setting following device extraction and infection resolution in the patients with prior infected ICDs. The defibrillation testing was effective, and there were no issues with S-ICD sensing. Furthermore, no complications or infections were reported during a median follow-up of 35 months. The authors concluded that simultaneous implantation of an S-ICD and leadless pacemaker is feasible and safe in patients at an elevated risk of infection requiring both ICD and pacing therapy.^36^

It is important to highlight that, once an S-ICD is paired with a concomitant LP system, the system loses some of its safety advantages pertaining to the risks of implanting an LP system—specifically, vascular injury, cardiac perforation, and tamponade. In addition, there are some valid concerns associated with the use of both devices together, as the effect of pacing on the sensing process of the S-ICD is not well studied. Previous investigations have demonstrated that pacing changes the morphology and amplitudes of different electrocardiogram components. Even when a patient passes S-ICD screening, it is possible that paced rhythm can affect the sensing capabilities of a concomitantly implanted S-ICD. In this case, there is a potential risk of inappropriate shock due to oversensing of the paced rhythm. We have previously demonstrated through a pilot study that it is at least theoretically feasible in most patients to concomitantly use both devices if we adopt a personalized device therapy approach, where the implantation procedure for the devices and their respective programming need to be tailored for every individual patient.^37^

Current guidelines recommend routine defibrillation testing in recipients of S-ICDs unless there is a clear contraindication.^38^ This is particularly important in the presence of a concomitant pacemaker, whether leadless or traditional. This is to ensure that the S-ICD does not inadvertently withhold therapy in the event of ventricular fibrillation after misinterpreting pacing spikes as normal ventricular activity.

In medical practice, it is imperative that the benefits are weighed against the risks associated with any treatment, and this case is not an exception. Cardiologists need to individualize the risk of SCD in cardiac sarcoidosis patients against the risks and complications associated with defibrillation protection device therapy. Further studies are required to evaluate the safety and efficacy of combining LP with S-ICD in human subjects. We believe that cardiac sarcoidosis patients, who could not benefit from the recent advances in device therapy before, have the potential to benefit the most from such a new concept of combined device therapy with mitigation of some of the risks associated with traditional device therapy. This possible shift in the risks associated has the potential to tip the balance toward offering more patients a potentially lifesaving therapy that is currently withheld owing to the risk of the traditional therapy itself.

As such, we strongly advocate considering this population of patients in clinical trials that investigate the efficacy and safety of this combined device therapy in human subjects. Further evidence is much needed to guide management of the risk of SCD in this population.

## Conclusion

Cardiac sarcoidosis remains an enigmatic health condition to general health practitioners and cardiologists alike. There is still a high burden of SCD owing to ventricular arrhythmias despite established guidance on the use of cardiac defibrillator therapy in this cohort of patients.

Cardiologists currently face complex decisions balancing between the risk of SCD and the risk of defibrillator-related complications in this special cohort of patients. The idea of combining LP therapy with S-ICD protection provides an exciting alternative therapy option that offers advantages over traditional TV therapy. This might eventually tip the balance toward greater use of defibrillator protection following a shared decision-making process in this special cohort.

## Figures and Tables

**Table 1:** Current Guideline Recommendations for an Implantable Cardioverter-defibrillator in Patients with Cardiac Sarcoidosis

Criteria	AHA/ACC/HRS 2017	ESC 2022
Prior aborted cardiac arrest in cardiac sarcoidosis	Class I	Class I
Documented spontaneous sustained VT in cardiac sarcoidosis	Class I	Class I
LVEF < 35% in cardiac sarcoidosis	Class I	Class I
LVEF > 35% with an indication for a permanent pacemaker in cardiac sarcoidosis	Class IIa	Class IIa
History of syncope with an arrhythmogenic cause in cardiac sarcoidosis	Class IIa	Class IIa
Induction of sustained VT during an EP study in cardiac sarcoidosis	Class IIa	Class IIa (LVEF 35%–50% with minor LGE)
LVEF > 35% with myocardial scar on MRI/PET in cardiac sarcoidosis	Class IIa	Class IIa (significant LGE after resolution of acute inflammation)
Active inflammation on PET in cardiac sarcoidosis	Not specifically addressed	Not specifically addressed

*Abbreviations:* ACC, American College of Cardiology; AHA, American Heart Association; EP, electrophysiology; ESC, European Society of Cardiology; HRS, Heart Rhythm Society; LGE, late gadolinium enhancement; LVEF, left ventricular ejection fraction; MRI, magnetic resonance imaging; PET, positron emission tomography; VT, ventricular tachycardia.
